# Soliton structures and dynamical characteristics of fractional nonlinear waves in the classical Boussinesq framework

**DOI:** 10.1038/s41598-026-37442-w

**Published:** 2026-02-07

**Authors:** Nasrin Nahar Rimu, Md. Antajul Islam, Pinakee Dey

**Affiliations:** Department of Mathematics, Mawlana Bhashani Science and Technology University, Tangail, 1902 Bangladesh

**Keywords:** Shallow water, Dynamical behavior, Chaotic interactions, Stability analysis, Sensitivity analysis, Plasma physics, Mathematics and computing, Physics

## Abstract

This paper investigates the time–space fractional classical Boussinesq equation, a nonlinear partial differential equation that describes long-wave propagation in shallow water. The modified extended tanh function formalism yields bright and dark solitons, breather-type waves, and periodic waves. The dynamical behavior of these solutions is revealed with bifurcation theory and phase-plane analysis: stable and unstable wave profiles change, and there may be chaotic interactions among them. The sensitivity analysis and the linear stability analysis have guaranteed the robustness of the solutions to small perturbations. The key results indicate that the equation facilitates a deep set of stable nonlinear wave forms, which exhibit predictable dynamical actions, to promote research into nonlinear fractional wave phenomena. Shallow water hydrodynamics, plasma physics, and nonlinear lattice systems can utilize these findings. The future research could take these findings to a new level by adding stochastic effects, fractional systems of higher dimensions, and numerical simulations to make the analysis and feasible validation more thorough.

## Introduction

Nonlinear evolution equations are central to the mathematical description of physically complex phenomena in many areas, such as fluid mechanics, plasma physics, and nonlinear lattices. The most notorious among these is the classical Boussinesq equation, utilized to describe long-wave propagation in shallow water and seen to be nonlinear and dispersive in nature^1–6^. In recent years, an interesting extension of the equation to fractional derivatives in space–time has received much interest because they have a better ability to describe the memory effects^7,8^ and the anomalous diffusion of real-world systems^9–12^. Quantitative complexity is further compounded by fractional formulation^13,14^, in which areas of both advanced analysis and numerical methods are needed to derive explicit solutions and gain insight into underlying dynamics^15–20^.

The space–time fractional classical Boussinesq equation is

1.1 $$D_{t}^{2\beta } u - c^{2} D_{x}^{2\alpha } u - aD_{x}^{4\alpha } u + bD_{x}^{2\alpha } \left( {u^{2} } \right) = 0,$$

where ${D}_{t}^{2\beta }u$ is a fractional acceleration of the wave with regard to time, ${c}^{2}{D}_{x}^{2\alpha }u$ is a model of fractional spatial propagation of wave speed $\omega$, $a{D}_{x}^{4\alpha }u$ is a higher-order dispersion effect, and $b{D}_{x}^{2\alpha }\left({u}^{2}\right)$ models the wave-wave interaction, which is nonlinear. The speed of the wave and the intensity of dispersion and nonlinearity are determined by the coefficients c, a, and b, respectively.

Optical fiber modeling with $\beta -$ fractional derivatives can provide a more versatile model for the complicated dynamics of light propagation, particularly in scenarios with anomalous dispersion and non-linear interactions. This model generalizes the nonlinear Schrodinger equation (NLSE) to the correct pulse dynamics in fiber with complex dispersion properties^21,22^. The combination of $\beta -$ fractional derivatives increase the accuracy of the models, making it possible to identify new effects and phenomena in fiber optics and improving the knowledge of light propagation in complex media. The $\beta -$ derivative of any function is defined as:

$${D}_{s}^{ \beta }m\left(s\right)=\underset{\delta \to \infty }{\mathrm{lim}}\frac{m\left(s+\delta {\left(s+\frac{1}{\Gamma \beta }\right)}^{1-\beta }\right) - m(s)}{\delta },$$

where $$0 <\beta \le 1$$, $$s>0$$.

The $\beta$-derivative meets some of the following properties:

1. $$D_{s}^{\beta } m\left( s \right) = \left( {s + \frac{1}{\Gamma \beta }} \right)^{1 - \beta } \frac{dm\left( s \right)}{{ds}}$$.
2. $$D_{s}^{\beta } \left( {\alpha u\left( s \right) + \gamma v\left( s \right)} \right) = \alpha D_{r}^{\beta } u\left( s \right) + \gamma D_{r}^{\beta } v\left( s \right)$$.
3. $$D_{s}^{\beta } \left( {u\left( s \right) vs} \right) = u\left( s \right) D_{r}^{\beta } v\left( s \right) + v\left( s \right) D_{r}^{\beta } u\left( s \right)$$.
4. $$D_{s}^{\beta } \left( {\frac{u\left( s \right)}{{v\left( s \right)}}} \right) = \frac{{v\left( s \right) D_{r}^{\beta } u\left( s \right) - u\left( s \right) D_{r}^{\beta } v\left( s \right)}}{{v^{2} \left( s \right)}}$$.
5. $D_{s}^{\beta } l = 0$, where l denotes a constant.

Study of the Boussinesq equation and variants has given rise over the years to many analytical and numerical methods. In recent decades, many different analytical approaches have been used to find exact and approximate solutions to NLEEs. They are the $(G{\prime} /G)-$ expansion technique^23^, the $(G{\prime} /G, 1/G)-$ expansion technique^24–28^, extended tanh function method^29,30^, the sine–cosine method^15,31^, Jacobi elliptic functions methods^32–35^, the Hirota bilinear method^36–38^, Darboux and Backlund transformations^39–42^, the Kudryashov method^43,44^, Modified extended direct algebraic method^45–49^, the Exp-function method^50–53^, and Lie symmetry^54–56^. These methods have all their merits, but the modified extended tanh function technique provides greater flexibility and versatility when applied to fractional equations so that we can now get both localized and periodic nonlinear structures in this manner.

The adequate analytical methods, e.g., the modified extended tanh function technique, have been shown to be extremely efficient in the derivation of the exact solutions of nonlinear fractional differential equations. The method allows one to form various soliton structures, such as bright and dark solitons, breather type, periodic waves, and $\mu -$ type soliton, and provides more insight into the nature of propagation properties of fractional nonlinear waves. In addition to these analytical solutions, analysis of bifurcation phenomena using a phase-plane, exploration of chaotic dynamics, and sensitivity analysis cast a light on complex couplings and parameter sensitivity in the system as well. To clearly illustrate the influence of the fractional derivative on wave propagation dynamics, we present the solution profiles using 2D plots rather than 3D visualizations. This approach enhances the interpretability of amplitude variations, phase-plane behavior, and sensitivity to parameter changes, as recommended in recent studies on fractional nonlinear systems^57–59^. Recent investigations into fractional dispersive systems have further highlighted their complex nonlinear dynamics, with Alaoui et al. (2025) demonstrating modulation instability and bifurcation structures in fractional equations, findings that closely align with our analysis of soliton stability and dynamical behavior in the fractional Boussinesq framework^60^.

Another important point is stability analysis, which confirms how the acquired soliton solutions resist infinitesimal perturbations and makes the theoretical results physically significant. Recently, it was established that Boussinesq-type equations also have a natural bi-directional propagation, having forward- and backward-moving wave modes. This property is entirely consistent with our analysis of the fractional classical Boussinesq equation, in which the soliton and periodic structures derived propagate in one or both directions depending on which parameters to use^12,61^. Together with these approaches, a holistic framework arising through the picking up of these approaches can unite the construction of analysis solutions to the dynamical system theory, providing the qualitative and quantitative viewpoints in the space–time fractional classical Boussinesq equation.

The original contribution of the current work is that it uses all the presented techniques (the modified extended tanh function, bifurcation analysis, phase-plane methods, chaos and sensitivity analysis, and linear stability analysis) simultaneously on a fractional nonlinear wave equation. The approach here not only expands the currently known solution space of the classical Boussinesq equation but also offers a methodological survey of the dynamical properties, about which very little was said in previous work. This work has the original contribution of applying several techniques at once the modified extended tanh function, bifurcation analysis, phase-plane methods, chaos and sensitivity analysis, and linear stability analysis to a fractional nonlinear wave equation. This method is not only able to enlarge the known solution space of the classical Boussinesq equation but also offers a survey of the methodology of dynamical properties of the classical Boussinesq equation, which had been barely addressed in earlier research. The implications of these findings have high practical value, as they can be used to predict waves, currents, and floods in shallow waters to achieve improved water management and flood prevention; to investigate the dynamics of plasma and assist in the development of fusion technology; and to examine soliton propagation, particle interactions, and energy transfer in nonlinear lattice models to benefit materials design and energy systems. These applications emphasize the theoretical as well as practical applicability of the study.

Overall, this work characterizes the space–time fractional classical Boussinesq equation in terms of a coherent analysis and dynamical analysis of novel soliton solutions, the stability properties, bifurcation behavior, and chaotic dynamics, thus providing the explicit reference framework of future studies on fractional models in nonlinear waves.

The remainder of this work is structured as follows.

Section “Theoretical Procedure Framework” includes the theoretical procedure framework, the mathematical formulation of the problem, and the preparation of a basic scheme for the analytical work conducted in this study. Section “Conceptual computation and solutions” gives the conceptual calculation and detailed resolutions used in the modeled fractional-order system. In Section “Effect of fractional orders” we focus on the role of fractional order, discussing how the aspect of memory and nonlocality affects responses of soliton solutions. Section “Physical significance of solitons” indicates the physical importance of the achieved solitons, explaining how they can be applied to nonlinear wave propagation and real-life applications. The 6th section entails analysis of stability so that stability is developed in the derived solutions. Section “Discussion of equilibrium states and bifurcations” and “Chaotic behavior” refers to the states of equilibrium, bifurcations, and chaos born in the system. Section “Sensitivity Analysis” is devoted to sensitivity analysis, indicating how sensitive the system is under parameter and initial conditions. Section “Lyapunov stability” uses the theory of Lyapunov stability to distinguish between stable and unstable regimes. Section “Novelty” denotes the novelty of the current study, whereas Section “Conclusion” concludes the paper, illustrating a summary of the key results and proposing the directions of future research.

## Theoretical procedure framework

In this section, the systematic procedure of deriving correct solutions of the solitons of FNLEEs is studied. We will begin by discussing a nonlinear evolution equation presented in the following form

2.1 $$S\left( {u,D_{t}^{\beta } u,D_{x}^{\alpha } u,D_{t}^{2\beta } u,D_{x}^{2\alpha } u,D_{x}^{3\alpha } u, \ldots } \right) = 0,$$

here, $u = u\left( {x_{i} , t} \right); \left( {i = 1,2,3,4, \ldots } \right)$ is a spatio-temporal wave function, and this equation may have fractional derivatives of the wave function $u\left( {x_{i} , t} \right)$. A wave transformation and nonlinear ordinary differential equation are developed to decrease one dimensionality. The transformation employed is

2.2 $$u\left( {x,t} \right) = u\left( \xi \right),$$

where $\xi =\frac{k}{\alpha }{(x+\frac{1}{\Gamma \alpha })}^{\alpha }-\frac{\omega }{\beta }{(t+\frac{1}{\Gamma \beta })}^{\beta }$, $\omega$ denotes the speed of the wave. The corresponding ordinary differential equation is articulated as

2.3 $$S\left( {u,u^{\prime},u^{\prime},u^{\prime\prime},u^{\prime\prime},u^{\prime},u^{\prime\prime\prime}, \ldots } \right) = 0,$$

This process is accomplished by taking the $\beta -$ fractional derivative, the definition and properties of which have been presented in the Introduction, and its linearity, product, and quotient rules to convert Eq. (2.2) to the form of Eq. (2.3).

where the prime indicates the order of the differential equation of the new wave function within its domain $\xi$. Similar to alternative approaches to analyze the problem analytically, the modified extended $tanh$ function technique examines a solution of series type to the transformed ordinary differential equation^62^. A planar resolution to this equation is provided by

2.4 $$u\left( \xi \right) = a_{0} + \mathop \sum \limits_{n = 0}^{M} a_{i} F^{i} \left( \xi \right) + \mathop \sum \limits_{n = 1}^{M} b_{i} F^{ - i} \left( \xi \right).$$

The solution integrates multiple arbitrary constants ${a}_{i}$, ${b}_{i}$, and a function $F(\xi )$, which is derived as an exhaustive solution to the Riccati problem.

2.5 $$F^{\prime}\left( \xi \right) = q + F^{2} \left( \xi \right).$$

The Riccati equation generates many generic solutions, depending on the given limitation of the free parameter $q$. The growth in (2.4) can also be thought of as a rational growth of the solution to the Riccati Eq. (2.5), and hence the applicability of the modified rational function method^63,64^. The Riccati equation generates many standardized solutions that are functions of the value of the parameter $q$.

*Category 1*: For $q\ne 0$, Eq. (2.5) designated a resolution as.

(Case 1): If $q<0$, then

$$F\left( \xi \right) = \left\{ {\begin{array}{*{20}c} { - \sqrt { - q} \tanh \left( {\sqrt { - q} \xi } \right)} \\ { - \sqrt { - q} \coth \left( {\sqrt { - q} \xi } \right)} \\ \end{array} } \right.$$

(Case 2): If $q>0$, then

$$F\left( \xi \right) = \left\{ {\begin{array}{*{20}c} {\sqrt q \tan \left( {\sqrt q \xi } \right)} \\ { - \sqrt q \cot \left( {\sqrt q \xi } \right)} \\ \end{array} } \right.$$

*Category 2*: If $q=0$, then

$$F\left( \xi \right) = - \frac{1}{\xi }$$

By substituting (2.4) and (2.5) into (2.3), a system of equations is generated, and solving it yields the values of the constants.

The reason we selected the modified extended function formalism is that it provides a systematic way to build up exact solutions to nonlinear equations, and it can also represent a very broad range of types of solutions. This method is simpler and more direct than some of the other methods, such as the Hirota bilinear method or Darboux transformations, which have to present explicit analytical solutions. Adding this explanation would highlight the innovative nature of our method and would also support the methodological soundness of our research. Here, we discuss the theoretical framework of the fractional Boussinesq equation and derive the principal equations and constraints. Section “Conceptual computation and solutions” further develops this by deriving the necessary expressions to find the exact solutions, like solitons, breathers, and periodic waves. The computational methods used in Section “Conceptual computation and solutions” to come up with precise solutions are based on the theoretical framework developed in Section “Theoretical Procedure Framework”. The structure facilitates an easy and rational movement in between the sections.

## Conceptual computation and solutions

**Fig. 1:**
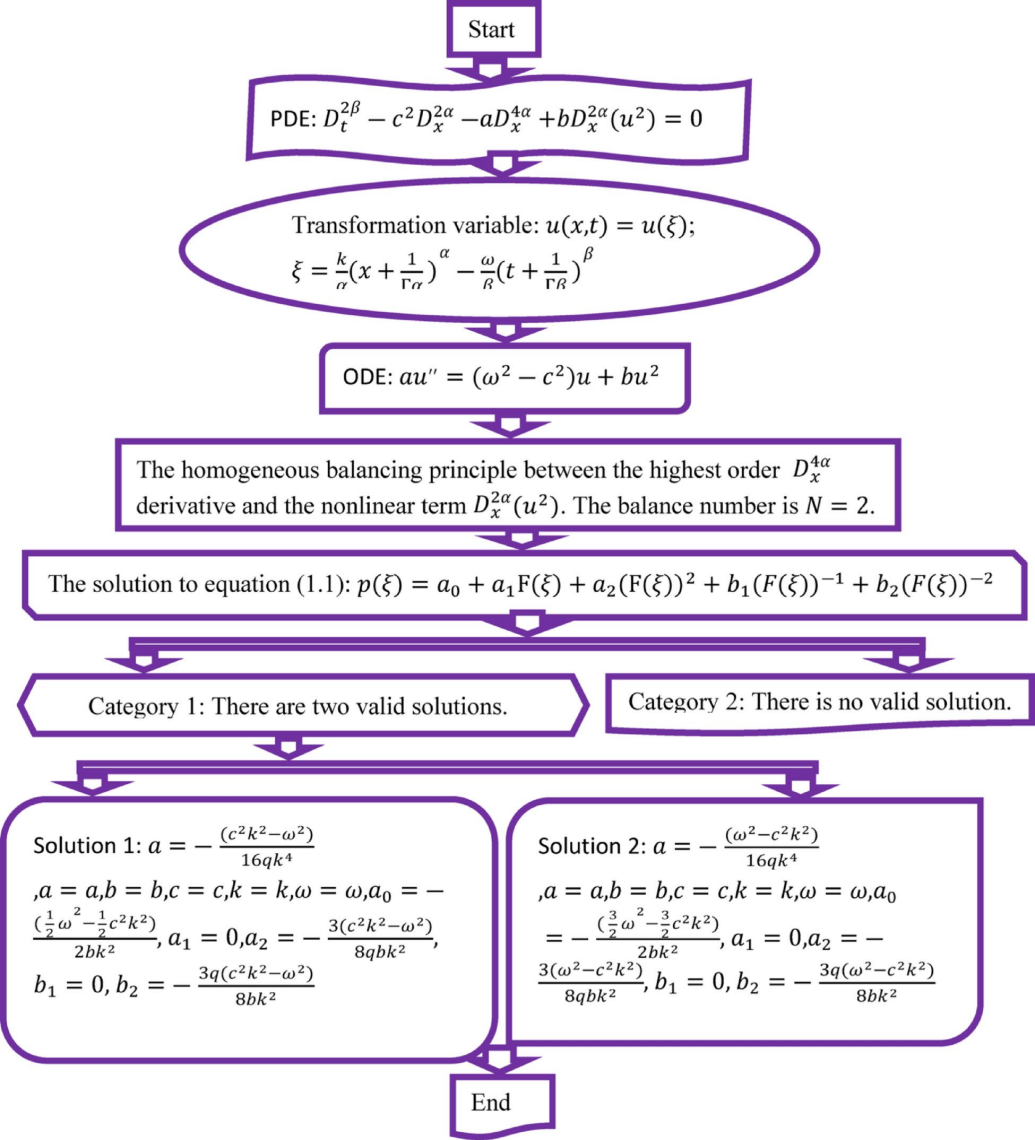
Flowchart depicting the theoretical estimation and resolution procedure.

Figure 1 appears as,

By letting $q \ne 0$, the universal solution of the Riccati problem, including hyperbolic and trigonometric functions, is then obtained, and the hyperbolic solution found by setting positive $q < 0$ is obtained by putting the above values of the parameters.

For solution 1:

3.1 $$\begin{aligned} u\left( \xi \right) = & - \frac{{\left( {\frac{1}{2}\omega^{2} - \frac{1}{2}c^{2} k^{2} } \right)}}{{2bk^{2} }} - \frac{{3\left( {c^{2} k^{2} - \omega^{2} } \right)}}{{8qbk^{2} }}\left( { - \sqrt { - q} \tanh \left( {\sqrt { - q} \xi } \right)} \right)^{2} \\ & - \frac{{3q\left( {c^{2} k^{2} - \omega^{2} } \right)}}{{8bk^{2} }}\left( { - \sqrt { - q} \tanh \left( {\sqrt { - q} \xi } \right)} \right)^{ - 2} . \\ \end{aligned}$$

and

3.2 $$\begin{aligned} u\left( \xi \right) = & - \frac{{\left( {\frac{1}{2}\omega^{2} - \frac{1}{2}c^{2} k^{2} } \right)}}{{2bk^{2} }} - \frac{{3\left( {c^{2} k^{2} - \omega^{2} } \right)}}{{8qbk^{2} }}\left( { - \sqrt { - q} \coth \left( {\sqrt { - q} \xi } \right)} \right)^{2} \\ & - \frac{{3q\left( {c^{2} k^{2} - \omega^{2} } \right)}}{{8bk^{2} }}\left( { - \sqrt { - q} \coth \left( {\sqrt { - q} \xi } \right)} \right)^{ - 2} . \\ \end{aligned}$$

where $\xi = \frac{k}{\alpha }\left( {x + \frac{1}{{{\Gamma }\alpha }}} \right)^{\alpha } - \frac{\omega }{\beta }\left( {t + \frac{1}{{{\Gamma }\beta }}} \right)^{\beta }$ & $u\left( {x,t} \right) = u\left( \xi \right)$, we get form (3.1) & (3.2)

3.3 $$\begin{aligned} u_{1} \left( {x,t} \right) = & - \frac{{(\frac{1}{2}\omega^{2} - \frac{1}{2}c^{2} k^{2} )}}{{2bk^{2} }} - \frac{{3\left( {c^{2} k^{2} - \omega^{2} } \right)}}{{8qbk^{2} }}\left( { - \sqrt { - q} \tanh \left( {\sqrt { - q} \left( {\frac{k}{\alpha }\left( {x + \frac{1}{{{\Gamma }\alpha }}} \right)^{\alpha } - \frac{\omega }{\beta }\left( {t + \frac{1}{{{\Gamma }\beta }}} \right)^{\beta } } \right)} \right)^{2} } \right) \\ & - \frac{{3q\left( {c^{2} k^{2} - \omega^{2} } \right)}}{{8bk^{2} }}\left( { - \sqrt { - q} \tanh \left( {\sqrt { - q} \left( {\frac{k}{\alpha }\left( {x + \frac{1}{{{\Gamma }\alpha }}} \right)^{\alpha } - \frac{\omega }{\beta }\left( {t + \frac{1}{{{\Gamma }\beta }}} \right)^{\beta } } \right)} \right)} \right)^{ - 2} . \\ \end{aligned}$$

and

3.4 $$\begin{aligned} u_{2} \left( {x,t} \right) = & - \frac{{\left( {\frac{1}{2}\omega^{2} - \frac{1}{2}c^{2} k^{2} } \right)}}{{2bk^{2} }} - \frac{{3\left( {c^{2} k^{2} - \omega^{2} } \right)}}{{8qbk^{2} }}\left( { - \sqrt { - q} \coth \left( {\sqrt { - q} \left( {\frac{k}{\alpha }\left( {x + \frac{1}{{{\Gamma }\alpha }}} \right)^{\alpha } - \frac{\omega }{\beta }\left( {t + \frac{1}{{{\Gamma }\beta }}} \right)^{\beta } } \right)} \right)} \right)^{2} \\ & - \frac{{3q\left( {c^{2} k^{2} - \omega^{2} } \right)}}{{8bk^{2} }}\left( { - \sqrt { - q} \coth \left( {\sqrt { - q} \left( {\frac{k}{\alpha }\left( {x + \frac{1}{{{\Gamma }\alpha }}} \right)^{\alpha } - \frac{\omega }{\beta }\left( {t + \frac{1}{{{\Gamma }\beta }}} \right)^{\beta } } \right)} \right)} \right)^{ - 2} . \\ \end{aligned}$$

For solution 2:

3.5 $$\begin{aligned} u_{3} \left( {x,t} \right) = & - \frac{{(\frac{3}{2}\omega^{2} - \frac{3}{2}c^{2} k^{2} )}}{{2bk^{2} }} - \frac{{3\left( {\omega^{2} - c^{2} k^{2} } \right)}}{{8qbk^{2} }}\left( { - \sqrt { - q} \tanh \left( {\sqrt { - q} \left( {\frac{k}{\alpha }\left( {x + \frac{1}{{{\Gamma }\alpha }}} \right)^{\alpha } - \frac{\omega }{\beta }\left( {t + \frac{1}{{{\Gamma }\beta }}} \right)^{\beta } } \right)} \right)} \right)^{2} \\ & - \frac{{3q\left( {\omega^{2} - c^{2} k^{2} } \right)}}{{8bk^{2} }}\left( { - \sqrt { - q} \tanh \left( {\sqrt { - q} \left( {\frac{k}{\alpha }\left( {x + \frac{1}{{{\Gamma }\alpha }}} \right)^{\alpha } - \frac{\omega }{\beta }\left( {t + \frac{1}{{{\Gamma }\beta }}} \right)^{\beta } } \right)} \right)} \right)^{ - 2} . \\ \end{aligned}$$

and

3.6 $$\begin{aligned} u_{4} \left( {x,t} \right) = & - \frac{{\left( {\frac{3}{2}\omega^{2} - \frac{3}{2}c^{2} k^{2} } \right)}}{{2bk^{2} }} - \frac{{3\left( {\omega^{2} - c^{2} k^{2} } \right)}}{{8qbk^{2} }}\left( { - \sqrt { - q} \coth \left( {\sqrt { - q} \left( {\frac{k}{\alpha }\left( {x + \frac{1}{{{\Gamma }\alpha }}} \right)^{\alpha } - \frac{\omega }{\beta }\left( {t + \frac{1}{{{\Gamma }\beta }}} \right)^{\beta } } \right)} \right)} \right)^{2} \\ & - \frac{{3q\left( {\omega^{2} - c^{2} k^{2} } \right)}}{{8bk^{2} }}\left( { - \sqrt { - q} \coth \left( {\sqrt { - q} \left( {\frac{k}{\alpha }\left( {x + \frac{1}{{{\Gamma }\alpha }}} \right)^{\alpha } - \frac{\omega }{\beta }\left( {t + \frac{1}{{{\Gamma }\beta }}} \right)^{\beta } } \right)} \right)} \right)^{ - 2} . \\ \end{aligned}$$

On the contrary, assuming $q > 0$, the solution can be articulated in trigonometric notation as.

For solution 1:

3.7 $$\begin{aligned} u_{5} \left( {x,t} \right) = & - \frac{{(\frac{1}{2}\omega^{2} - \frac{1}{2}c^{2} k^{2} )}}{{2bk^{2} }} - \frac{{3\left( {c^{2} k^{2} - \omega^{2} } \right)}}{{8qbk^{2} }}\left( {\sqrt q \tan \left( {\sqrt q \left( {\frac{k}{\alpha }\left( {x + \frac{1}{{{\Gamma }\alpha }}} \right)^{\alpha } - \frac{\omega }{\beta }\left( {t + \frac{1}{{{\Gamma }\beta }}} \right)^{\beta } } \right)} \right)} \right)^{2} \\ & - \frac{{3q\left( {c^{2} k^{2} - \omega^{2} } \right)}}{{8bk^{2} }}\left( {\sqrt q \tan \left( {\sqrt q \left( {\frac{k}{\alpha }\left( {x + \frac{1}{{{\Gamma }\alpha }}} \right)^{\alpha } - \frac{\omega }{\beta }\left( {t + \frac{1}{{{\Gamma }\beta }}} \right)^{\beta } } \right)} \right)} \right)^{ - 2} . \\ \end{aligned}$$

and

3.8 $$\begin{aligned} u_{6} \left( {x,t} \right) = & - \frac{{(\frac{1}{2}\omega^{2} - \frac{1}{2}c^{2} k^{2} )}}{{2bk^{2} }} - \frac{{3\left( {c^{2} k^{2} - \omega^{2} } \right)}}{{8qbk^{2} }}\left( { - \sqrt q \cot \left( {\sqrt q \left( {\frac{k}{\alpha }\left( {x + \frac{1}{{{\Gamma }\alpha }}} \right)^{\alpha } - \frac{\omega }{\beta }\left( {t + \frac{1}{{{\Gamma }\beta }}} \right)^{\beta } } \right)} \right)^{2} } \right) \\ & - \frac{{3q\left( {c^{2} k^{2} - \omega^{2} } \right)}}{{8bk^{2} }}\left( { - \sqrt q \cot \left( {\sqrt q \left( {\frac{k}{\alpha }\left( {x + \frac{1}{{{\Gamma }\alpha }}} \right)^{\alpha } - \frac{\omega }{\beta }\left( {t + \frac{1}{{{\Gamma }\beta }}} \right)^{\beta } } \right)} \right)} \right)^{ - 2} . \\ \end{aligned}$$

For solution 2:

3.9 $$\begin{aligned} u_{7} \left( {x,t} \right) = & - \frac{{(\frac{3}{2}\omega^{2} - \frac{3}{2}c^{2} k^{2} )}}{{2bk^{2} }} - \frac{{3\left( {\omega^{2} - c^{2} k^{2} } \right)}}{{8qbk^{2} }}\left( {\sqrt q \tan \left( {\sqrt q \left( {\frac{k}{\alpha }\left( {x + \frac{1}{{{\Gamma }\alpha }}} \right)^{\alpha } - \frac{\omega }{\beta }\left( {t + \frac{1}{{{\Gamma }\beta }}} \right)^{\beta } } \right)} \right)} \right)^{2} \\ & - \frac{{3q\left( {\omega^{2} - c^{2} k^{2} } \right)}}{{8bk^{2} }}\left( {\sqrt q \tan \left( {\sqrt q \left( {\frac{k}{\alpha }\left( {x + \frac{1}{{{\Gamma }\alpha }}} \right)^{\alpha } - \frac{\omega }{\beta }\left( {t + \frac{1}{{{\Gamma }\beta }}} \right)^{\beta } } \right)} \right)} \right)^{ - 2} . \\ \end{aligned}$$

and

3.10 $$\begin{aligned} u_{8} \left( {x,t} \right) = & - \frac{{(\frac{3}{2}\omega^{2} - \frac{3}{2}c^{2} k^{2} )}}{{2bk^{2} }} - \frac{{3\left( {\omega^{2} - c^{2} k^{2} } \right)}}{{8qbk^{2} }}\left( { - \sqrt q \cot \left( {\sqrt q \left( {\frac{k}{\alpha }\left( {x + \frac{1}{{{\Gamma }\alpha }}} \right)^{\alpha } - \frac{\omega }{\beta }\left( {t + \frac{1}{{{\Gamma }\beta }}} \right)^{\beta } } \right)} \right)^{2} } \right) \\ & - \frac{{3q\left( {\omega^{2} - c^{2} k^{2} } \right)}}{{8bk^{2} }}\left( { - \sqrt q \cot \left( {\sqrt q \left( {\frac{k}{\alpha }\left( {x + \frac{1}{{{\Gamma }\alpha }}} \right)^{\alpha } - \frac{\omega }{\beta }\left( {t + \frac{1}{{{\Gamma }\beta }}} \right)^{\beta } } \right)} \right)} \right)^{ - 2} . \\ \end{aligned}$$

## Effect of fractional orders

To examine solitons, the solution also presents standard data in the form of graphs, such as the true solution amplitudes and null solutions. It draws 2D and 3D plots, contour graphs, and amplitude profiles of how they dynamically behave, including frequencies, phase evolutions, and dynamics. This multidimensional characterization explains the dynamics of solitons and makes the solutions acquired clearer.

The parameters of $\alpha$ and $\beta$ regulate the spatial and temporal distortion of the patterns of signal waves, respectively. In the case of $\alpha =1$, a uniform wavelength is produced. The $\alpha$ modifies the fronts and bends waves when $\alpha >1$. When $0<\alpha <1$, profiles undergo flattening, contrast, and distortion. A frequency is guaranteed by $\beta =1$. When galileology is valued at $0<\beta <1$, the frequency of the wave will decline over time, while the value of $\beta >1$ will cause it to increase. It is the value of the coefficient $\alpha$ that predetermines the spatial variation of the sharpness of the wave structures, whereas the apparent speed and their temporal development are determined by the coefficient $\beta$.

**Fig. 2:**
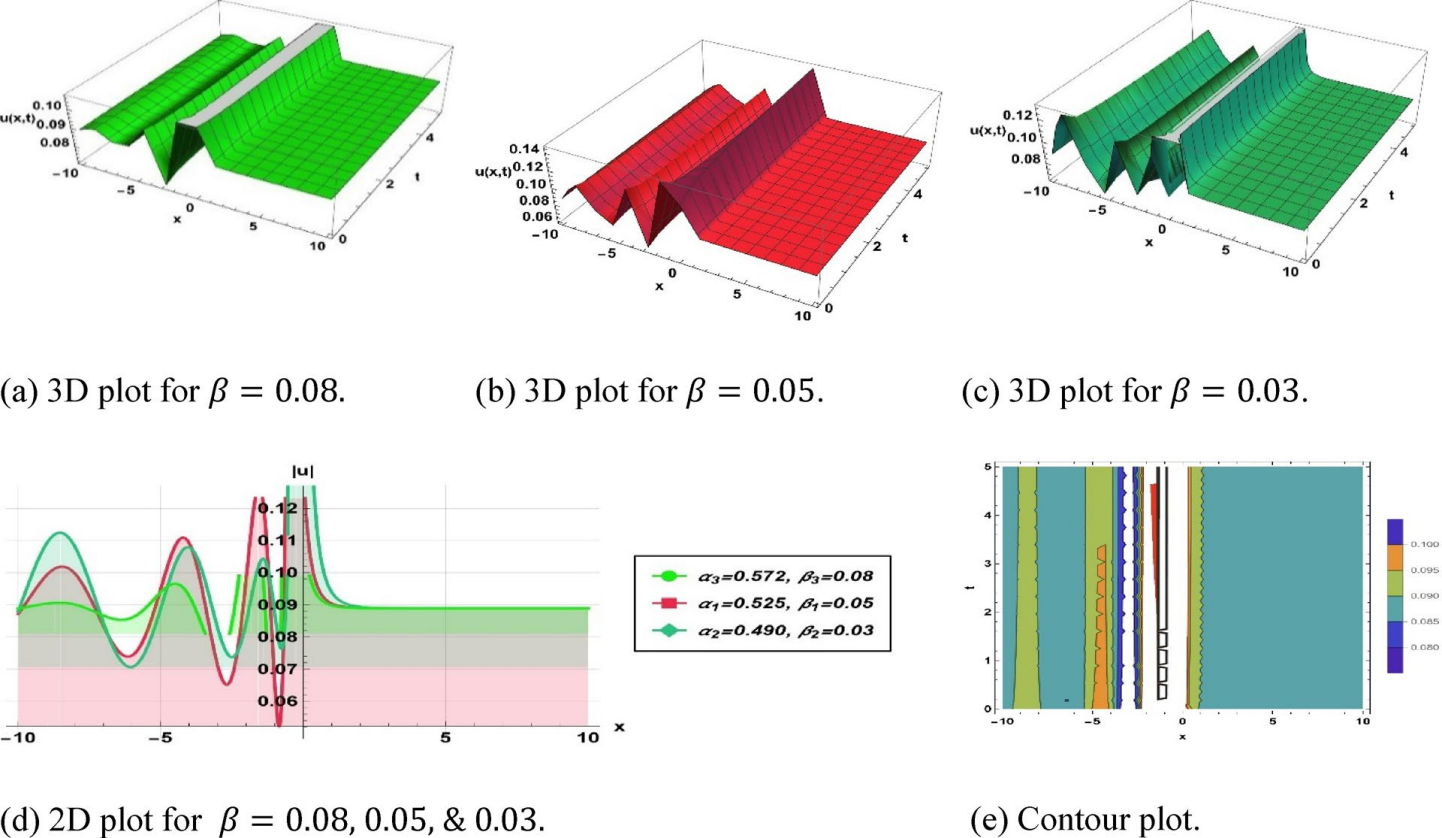
Parametric impact on bright type soliton to the Eq. (3.3) for different values.

Figure 2 illustrates the solution (3.3) as a bright type soliton, employing the parameter values $k=0.3, a=0.1, b=0.1, c=0.1,\omega =0.01,q=-7.77, {\alpha }_{1}=0.572, {\beta }_{1}=0.08, {\alpha }_{2}=0.525, {\beta }_{2}=0.05$ and ${\alpha }_{3}=0.49, {\beta }_{3}=0.03$.

**Fig. 3:**
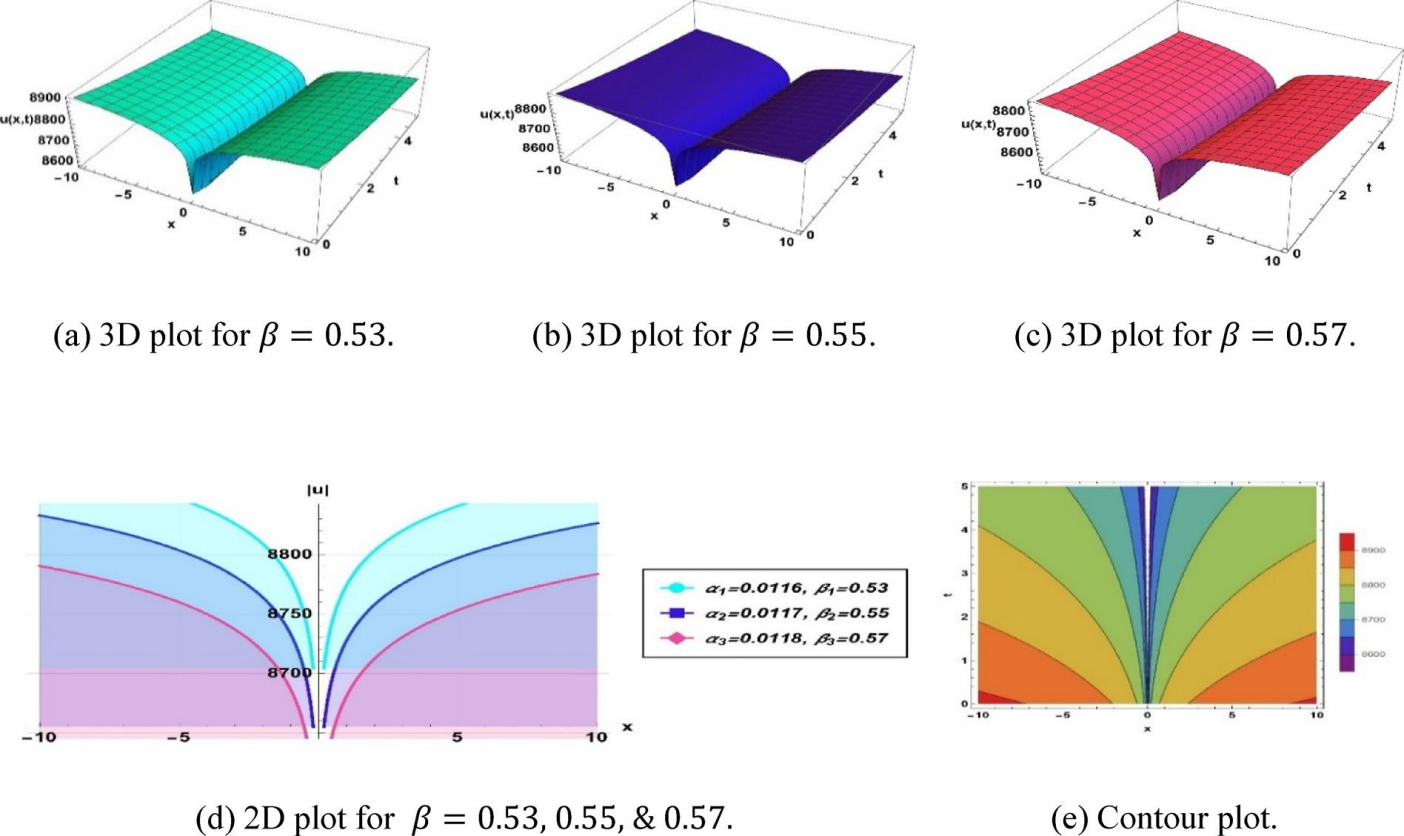
Parametric impact on dark type soliton to the Eq. (3.3) for different values.

The execution of the designated solution (3.3) utilizing parameters $k=0.01, a=2.46, b=0.1, c=1.1,\omega =0.01,q=-4.479, {\alpha }_{1}=0.0116, {\beta }_{1}=0.53, {\alpha }_{2}=0.0117, {\beta }_{2}=0.55$ and ${\alpha }_{3}=0.0118, {\beta }_{3}=0.57$, produces a dark type soliton, as illustrated in Fig. 3.

**Fig. 4:**
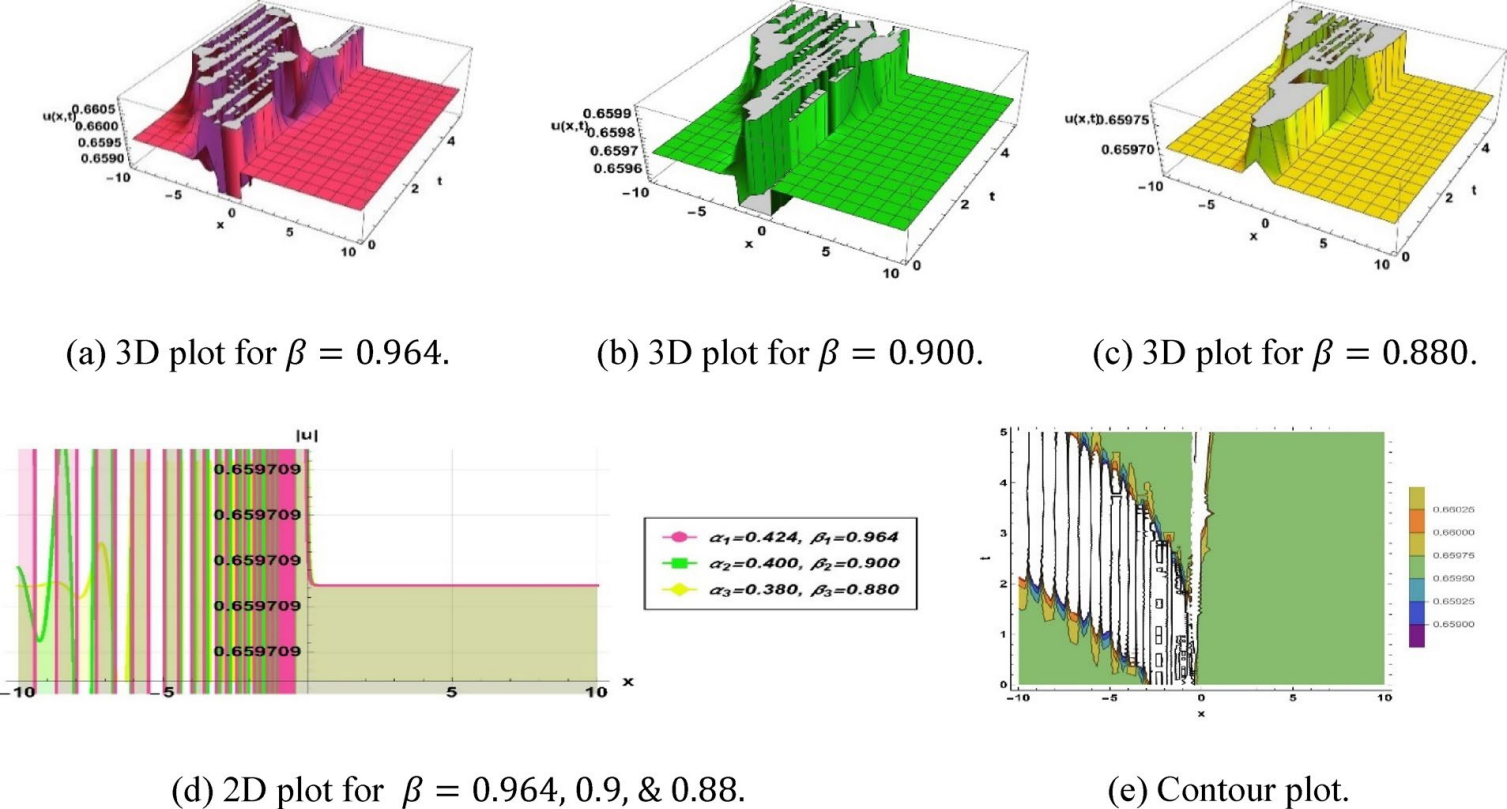
Parametric impact on breather type soliton to the Eq. (3.3) for different values.

Solution (3.3) yields a breather type soliton, seen in Fig. 4, defined by the parameters $k=3.64, a=5.819, b=9.26, c=3.14,\omega =1.1,q=-4.18, {\alpha }_{1}=0.424, {\beta }_{1}=0.964, {\alpha }_{2}=0.4, {\beta }_{2}=0.9$ and ${\alpha }_{3}=0.38, {\beta }_{3}=0.88$.

**Fig. 5:**
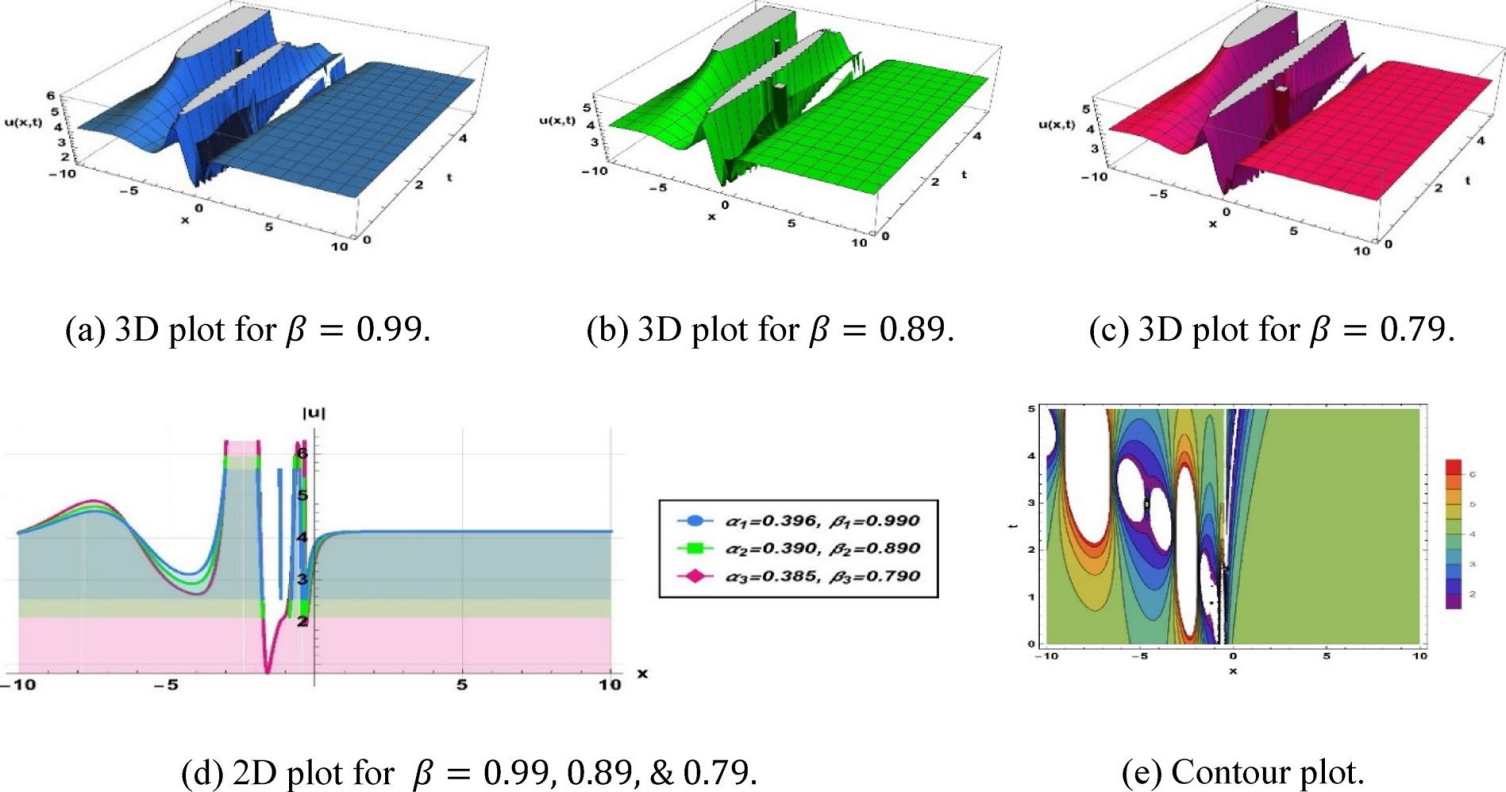
Parametric impact on breather type soliton to the Eq. (3.4) for different values.

The designated Eq. (3.4) illustrates a breather type soliton defined by $k=2.6, a=4.279, b=5.3, c=5.939,\omega =0.94,q=-0.329, {\alpha }_{1}=0.396, {\beta }_{1}=0.99, {\alpha }_{2}=0.390, {\beta }_{2}=0.89$ and ${\alpha }_{3}=0.385, {\beta }_{3}=0.79$, as shown in Fig. 5.

**Fig. 6:**
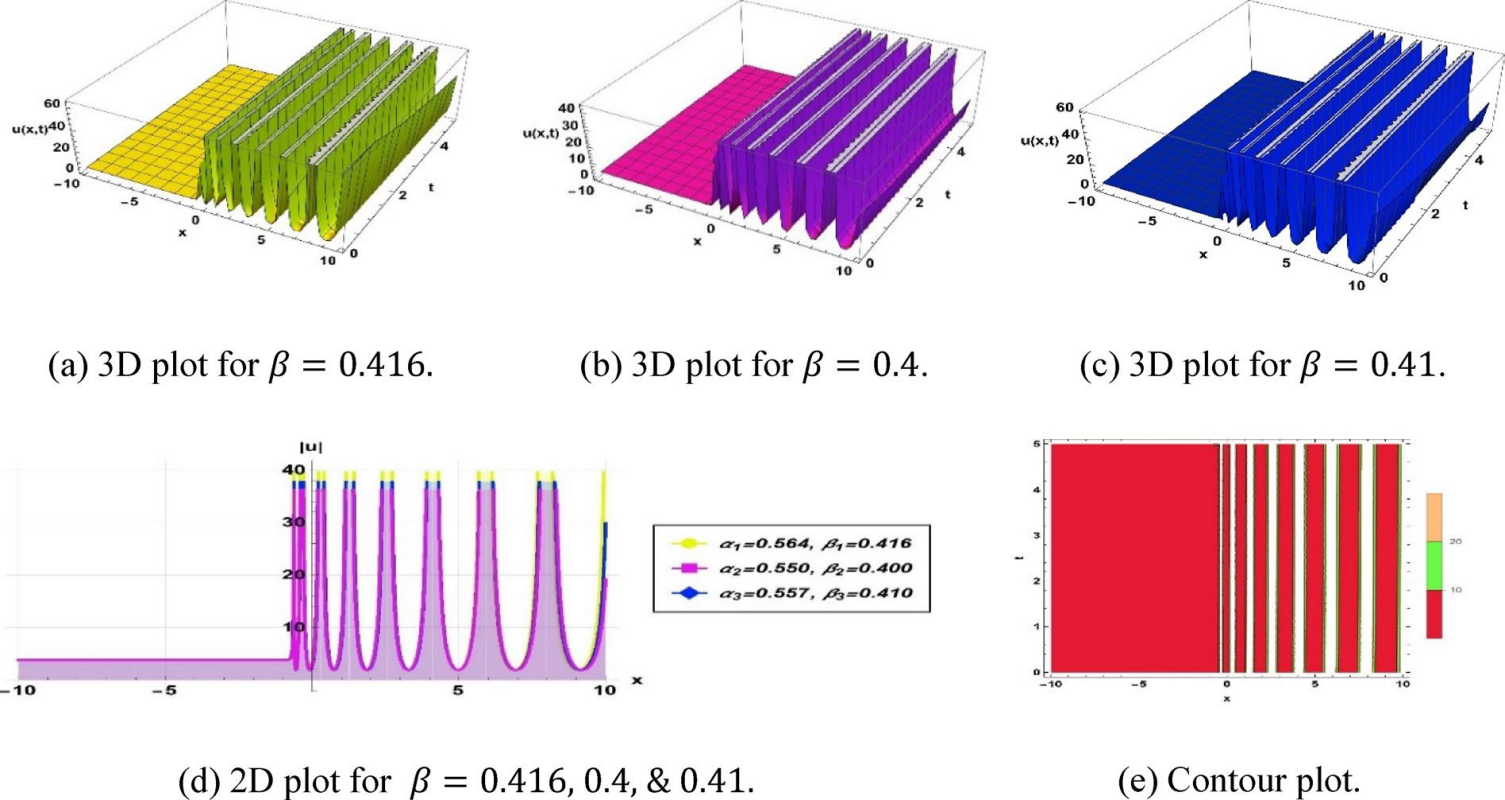
Parametric impact on periodic type soliton to the Eq. (3.7) for different values.

Solution (3.7) depicts a periodic type soliton, as illustrated in Fig. 6, with the parameters $k=1.1, a=0.1, b=3.1, c=3.44,\omega =0.01,q=2.84, {\alpha }_{1}=0.564, {\beta }_{1}=0.416, {\alpha }_{2}=0.55, {\beta }_{2}=0.4$ and ${\alpha }_{3}=0.557, {\beta }_{3}=0.41$.

**Fig. 7:**
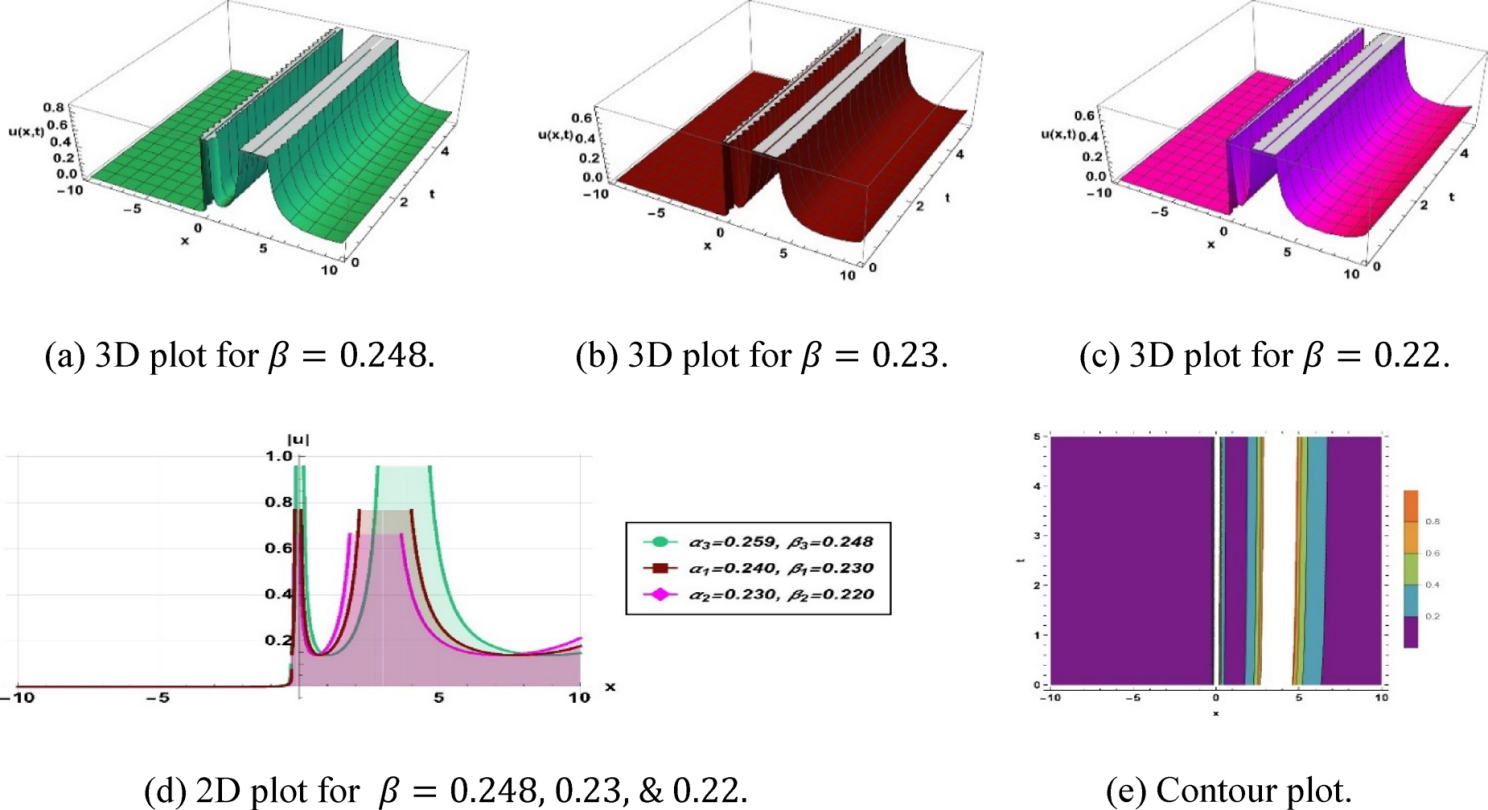
Parametric impact on $\mu$-type soliton to the Eq. (3.9) for different values.

Figure 7 depicts the $\mu$-type soliton obtained from solution (3.9), utilizing the parameters $k=0.48, a=0.1, b=0.1, c=0.1,\omega =0.01,q=1.48, {\alpha }_{1}=0.259, {\beta }_{1}=0.248, {\alpha }_{2}=0.24, {\beta }_{2}=0.23$ and ${\alpha }_{3}=0.23, {\beta }_{3}=0.22$.

**Fig. 8:**
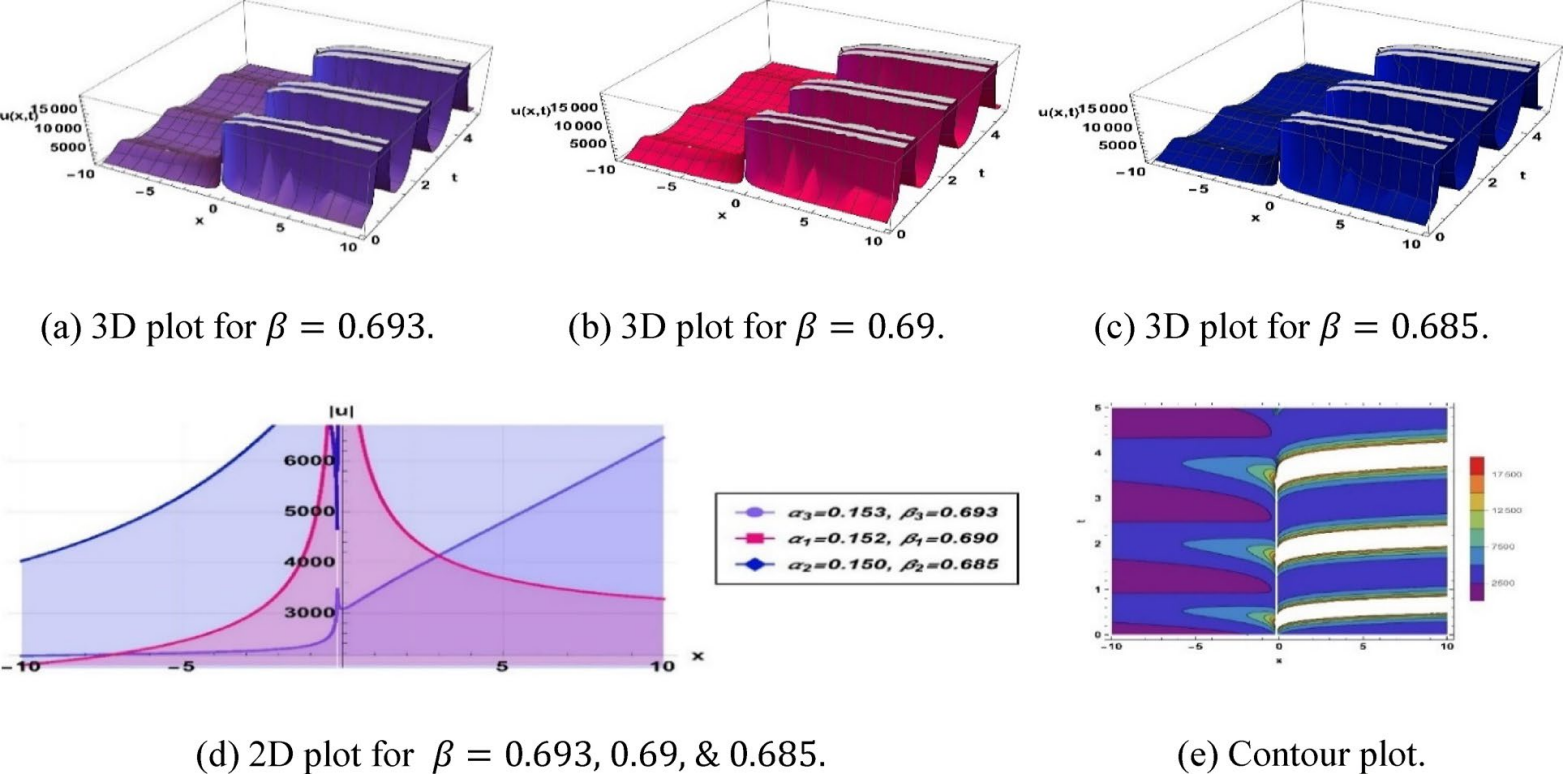
Parametric impact on breather type soliton to the Eq. (3.10) for different values.

Figure 8 depicts a breather type soliton obtained from the previously stated solution (3.10), dependent on the specific parameterization of $k=0.051, a=0.21, b=0.1, c=0.1,\omega =0.731,q=3.22, {\alpha }_{1}=0.153, {\beta }_{1}=0.693, {\alpha }_{2}=0.152, {\beta }_{2}=0.69$ and ${\alpha }_{3}=0.15, {\beta }_{3}=0.685$.

We showed mainly 3D plots, which better display the three-dimensionality of the effects of fractional derivatives on the propagation of the model. Moreover, we have added a 2D plot to give a clear image in a certain direction to empower the readers to compare it with much ease. The use of 3D and 2D plots will guarantee us the overall and clear representation of our results.

We examine the implications of the use of fractional-order derivatives on soliton behavior, especially memory effects, nonlocality, dynamics, and stability. The effects of memory make the system rely on the history of the system, altering the shape and amplitude of solitons, and nonlocality spreads interactions through an area, altering propagation and collisions. The issue of stability when small perturbations are introduced is investigated, and the effects of the fractional-order effects on stability are evaluated. These analyses are made possible through the use of the tool of fractional calculus that enables a better description of complex soliton phenomena.

## Physical significance of solitons

Nonlinear wave dynamics refers to various classes of so-called solitons, with their own physical meaning. Bright solitons are concentrations of energy in nonlinear media, whereas the dark solitons are dips or holes in a continuous background. Breather-like, that is, spatially localized, yet temporally oscillating structures, are responsible for creating rogue waves and modulation instability in optical and hydrodynamic systems. Periodic solitons such as cnoidal or dnoidal waves are nonlinear periodic wave trains that remain in balance in a repetitive form. Finally, in generalized models, opportunely called μ-type, there appear two parameters that govern the amplitude, width, and stability, depend on higher-order nonlinear corrections, and lead to different bifurcation modes or soliton families. The μ-type soliton form is a special solitary wave form that has a sharp localized peak and steep rising and falling edges and retains its shape as it propagates. In contrast to smooth bell-shaped solitons (e.g., KdV type), the μ-type soliton possesses a piecewise or step-like geometry with almost parallel smooth regions joined by sharp transitions. These soliton solutions provide examples of the varied forms of energy localization, stability, and nonlinear interaction in complex media.

## Stability analysis

**Fig. 9:**
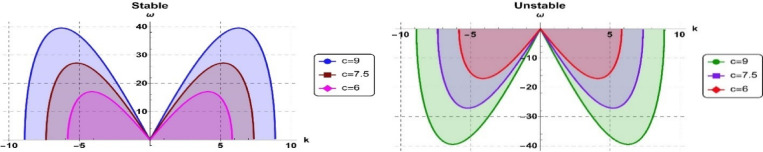
Visualization of stability analysis in 2D patterns to the Eq. (6.4) for different values.

Stability analysis Stability analysis employs a mathematical approach to ascertain the stability of a system in response to minor perturbations over time^27,65,66^. It facilitates the distinction between stable and unstable areas of operations and makes it predictable^67^. This subsection will discuss the stability nature of the classical Boussinesq equation with the help of a sophisticated method of examining the stability of the system, namely the response to infinitesimal perturbations, as shown in Fig. 9.

6.1 $$u\left( {x,t} \right) = U_{0} + \rho U\left( {x,t} \right).$$

The equilibrium state solution of the model, ${U}_{0}$ is a small constant, $\rho$ is a small constant. Whereas the perturbed wave solution, $u(x,t)$ is found by linearizing Eq. (1.1) and neglecting higher-order terms.

6.2 $$D_{t}^{2\beta } U - c^{2} D_{x}^{2\alpha } U - aD_{x}^{4\alpha } U + 2bU_{0} D_{x}^{2\alpha } U = 0.$$

The equation in discussion is a linear differential equation pertaining to the wave variable $U(x,t).$ The proposed answer for Eq. (6.2) can be articulated as follows:

6.3 $$U\left( {x,t} \right) = e^{{i\left( {\frac{k}{\alpha }\left( {x + \frac{1}{{{\Gamma }\alpha }}} \right)^{\alpha } - \frac{\omega }{\beta }\left( {t + \frac{1}{{{\Gamma }\beta }}} \right)^{\beta } } \right)}} .$$

Among the factors that affect the frequency of the wave are the wave number $k$, nonlinearity ($\alpha \& \beta$), and the phase velocity ($\omega$). By inserting the trial solution (6.3) into the Eq. (6.2), the dispersion relation is already obtained, it yields the phase velocity $\omega$.

$$\omega^{2} = k^{2} \left( {c^{2} - 2bU_{0} } \right) - ak^{4}$$

6.4 $$\Rightarrow \omega = \pm \sqrt {k^{2} \left( {c^{2} - 2bU_{0} } \right) - ak^{4} } .$$

In our current research, only deterministic perturbations have been taken into consideration. This reasoning is primarily because deterministic analysis makes a simply trackable route to fundamentally understand the dynamics and stability of the fractional Boussinesq equation in the absence of the complicating factor of random fluctuations. This basic knowledge is fundamental in the event of generalizing the work to more realistic stochastic perturbations. We do accept that stochastic effects are very applicable to real-world effects and could have a substantial impact on soliton dynamics and stability. The weakness of the current work is that the stochastic effects were not taken into account, and thus, the findings can only be applied to deterministic conditions.

## Discussion of equilibrium states and bifurcations

Bifurcation analysis is a method that converts fractional classical Boussinesq equations into planar dynamical systems, where nonlinearity and dispersion are included^60,66,68^. It enables systematic search in the conditions of the equilibrium states and local phase charts to observe the effects of fractional derivatives and control parameters on invariant surfaces and stability changes. Phase-plane provides a two-dimensional picture of phase-space structures that can reveal multi-stability, heteroclinic and homoclinic trajectories, and parameter-excited bifurcation^60,69^. The fractional classical Boussinesq equation is converted to a planar system by bifurcation analysis to understand the dependence of equilibrium states on a wide range of the fractional derivatives and control parameters. The 2D phase-plane analysis can display the system’s dynamics, identify multi-stability, oscillations, and bifurcations due to parameter variation, and help in understanding the stability of soliton, breather, and periodic wave solutions. The method is applicable to shallow water hydrodynamics, plasma physics, and nonlinear lattice structures. Let $\phi =u(x,t)$ and ${\phi }{\prime}=\psi$ then the dynamical system is

7.1 $$\left\{ {\begin{array}{*{20}c} {\phi^{\prime} = \psi } \\ {\psi^{\prime} = \frac{{\left( {\omega^{2} - c^{2} k^{2} } \right)\phi + bk^{2} \phi^{2} }}{{ak^{4} }}} \\ \end{array} } \right.$$

This system has a Hamiltonian structure as

7.2 $$H\left( {\phi ,\psi } \right) = \frac{{ak^{4} }}{2}\psi^{2} - \frac{{\left( {\omega^{2} - c^{2} k^{2} } \right)}}{2}\phi^{2} - \frac{{bk^{2} }}{3}\phi^{3} .$$

This system has three equilibrium points,

$$\left( {0,0} \right), \left( { - \frac{{\left( {\omega^{2} - c^{2} k^{2} } \right)}}{{bk^{2} }},0} \right).$$

Moreover, the Jacobian for the system is

$$J\left( {\phi ,\psi } \right) = \left| {\begin{array}{*{20}c} 0 & 1 \\ {\frac{{\left( {\omega^{2} - c^{2} k^{2} } \right)}}{{ak^{4} }} + \frac{2b}{{ak^{2} }}\phi } & 0 \\ \end{array} } \right|$$

The eigenvalues of the system under the equilibrium point $\left( {0,0} \right)$ are

$$\lambda = \pm \sqrt {\frac{{\left( {\omega^{2} - c^{2} k^{2} } \right)}}{{ak^{4} }}} .$$

A transcritical bifurcation is offered by the space–time fractional classical Boussinesq equation. A transcritical bifurcation is where two equilibriums collide and interchange stability, as a parameter is changed across a critical value. Such a bifurcation is typified by the presence of both a stable and an unstable steady state at the intersection point, and the stability of both branches’ changes. Transcritical bifurcations in nonlinear fractional systems (e.g., the Boussinesq model) are used to explain how changes in fractional order and system parameters determine the stability of waves and fluid transitions between different dynamic modes.

**Fig. 10:**
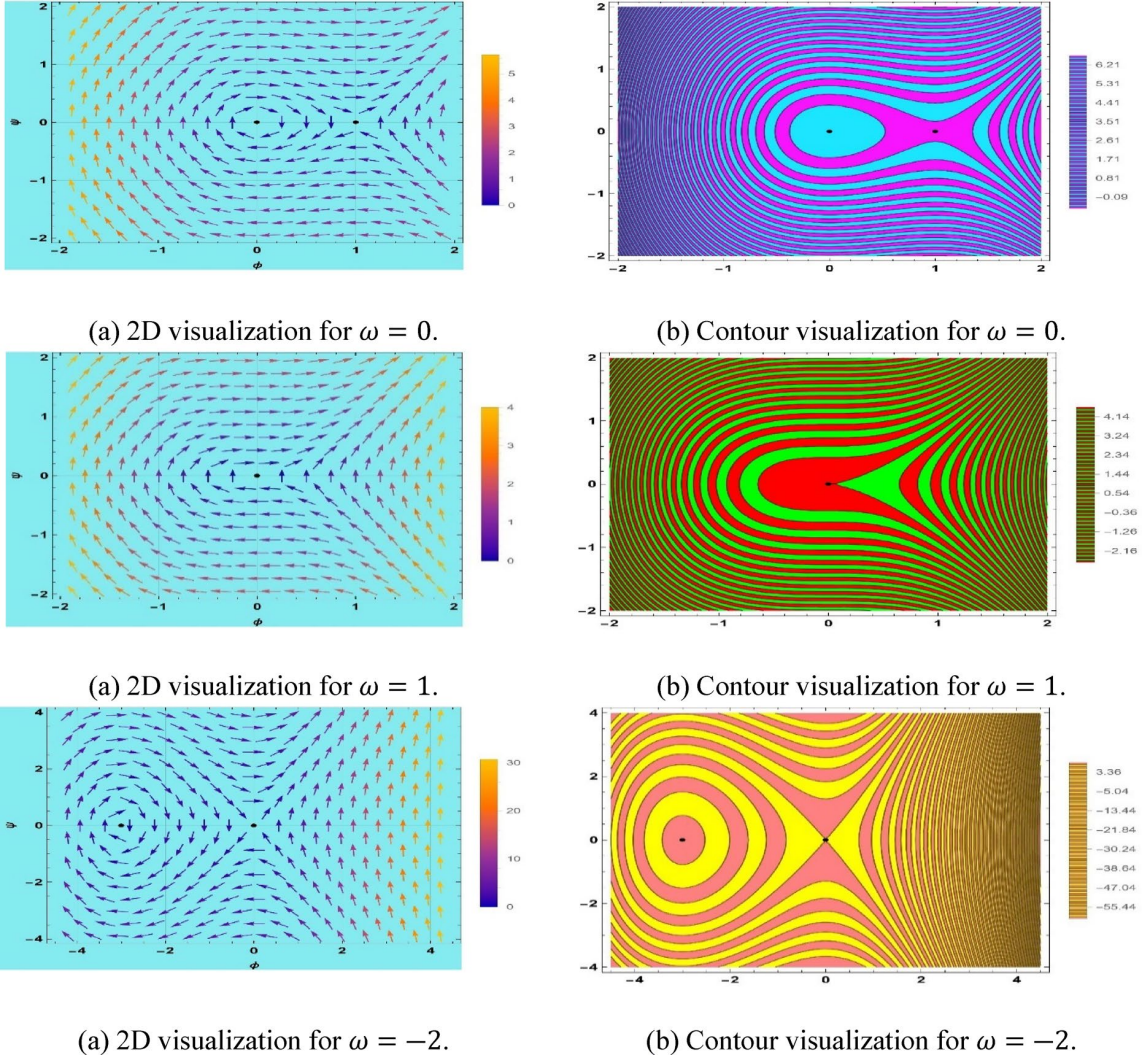
Representation of tanscritical bifurcation of system (7.1).

When $\omega =0$, two equilibrium points are identified at $(\mathrm{0,0})$ and $(\mathrm{1,0})$ , the point $(\mathrm{0,0})$ behaves as a stable center, while $(\mathrm{1,0})$ corresponds to an unstable saddle point. In the case of $\omega =1$, only a single equilibrium point, $(\mathrm{0,0})$ is obtained, and classified as a cuspidal point. Furthermore, when $\omega =-2$, two equilibrium points emerge at $(-\mathrm{3,0})$, and $(\mathrm{0,0})$, where $(-\mathrm{3,0})$ functions as a stable center and $(\mathrm{0,0})$ represents an unstable saddle point. All of these are represented in Fig. 10.

## Chaotic behavior

Chaotic systems are very sensitive to initial conditions such that there are dramatic variations in long-term behavior^60,70^. Numerous natural and man-made systems canvass fluids, optical systems, atmospheric processes, and in vitro technologies, including “organs on a chip,” to illustrate their occurrence. This study investigates the chaotic phenomenon associated with the space–time fractional classical Boussinesq equation by analyzing the effects of modifying the frequency and amplitude parameters. The coupled differential equations are obtained as a derivation of Eq. (7.1).

8.1 $$\left\{ {\begin{array}{*{20}c} {\phi^{\prime} = \psi } \\ {\psi^{\prime} = \frac{{\left( {\omega^{2} - c^{2} k^{2} } \right)\phi + bk^{2} \phi^{2} }}{{ak^{4} }} + \gamma cos\left( {\eta t} \right)} \\ \end{array} } \right.$$

**Fig. 11:**
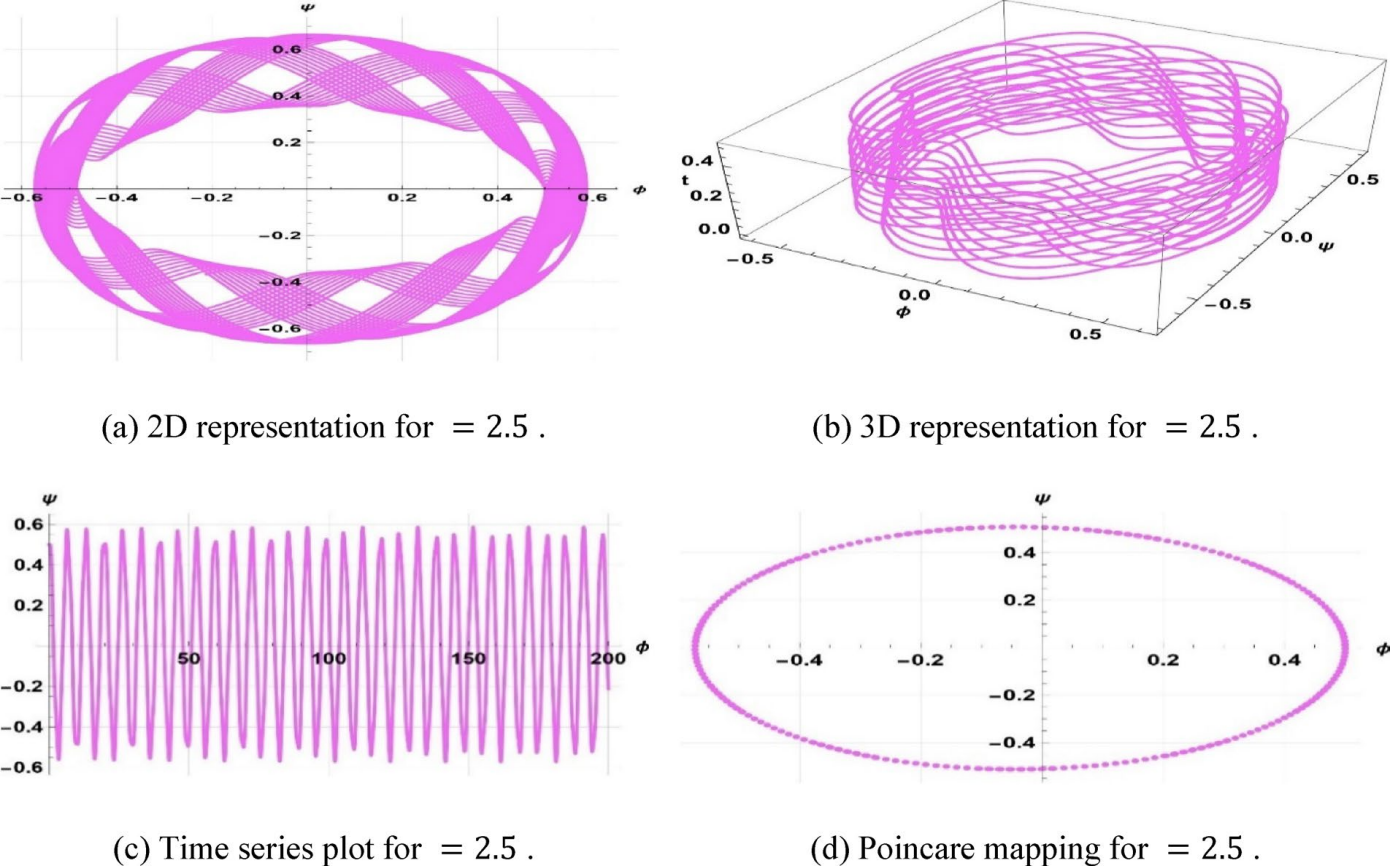
Illustration of periodic behaviors of system (8.1).

**Fig. 12:**
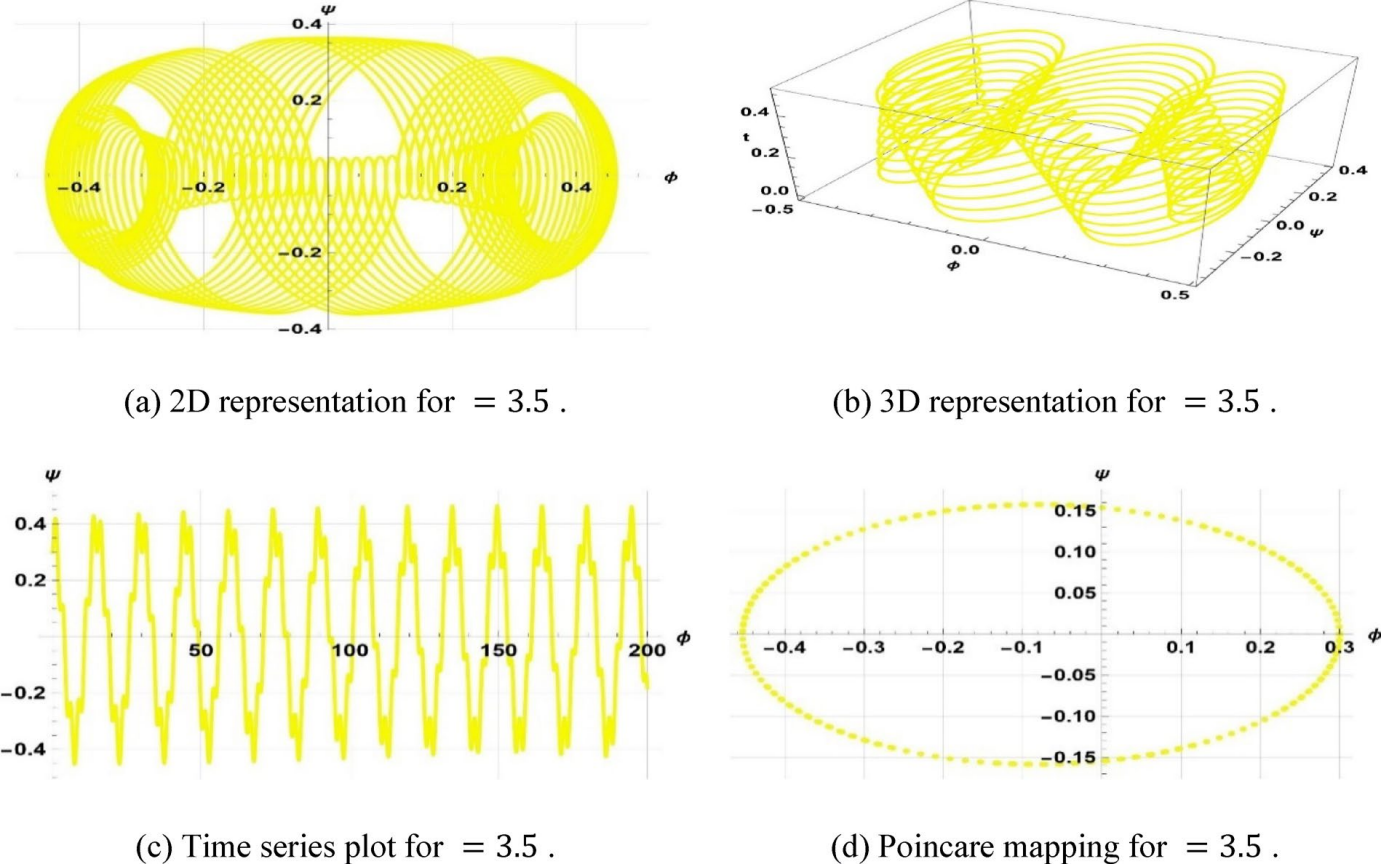
Illustration of quasi-periodic behaviors of system (8.1).

**Fig. 13:**
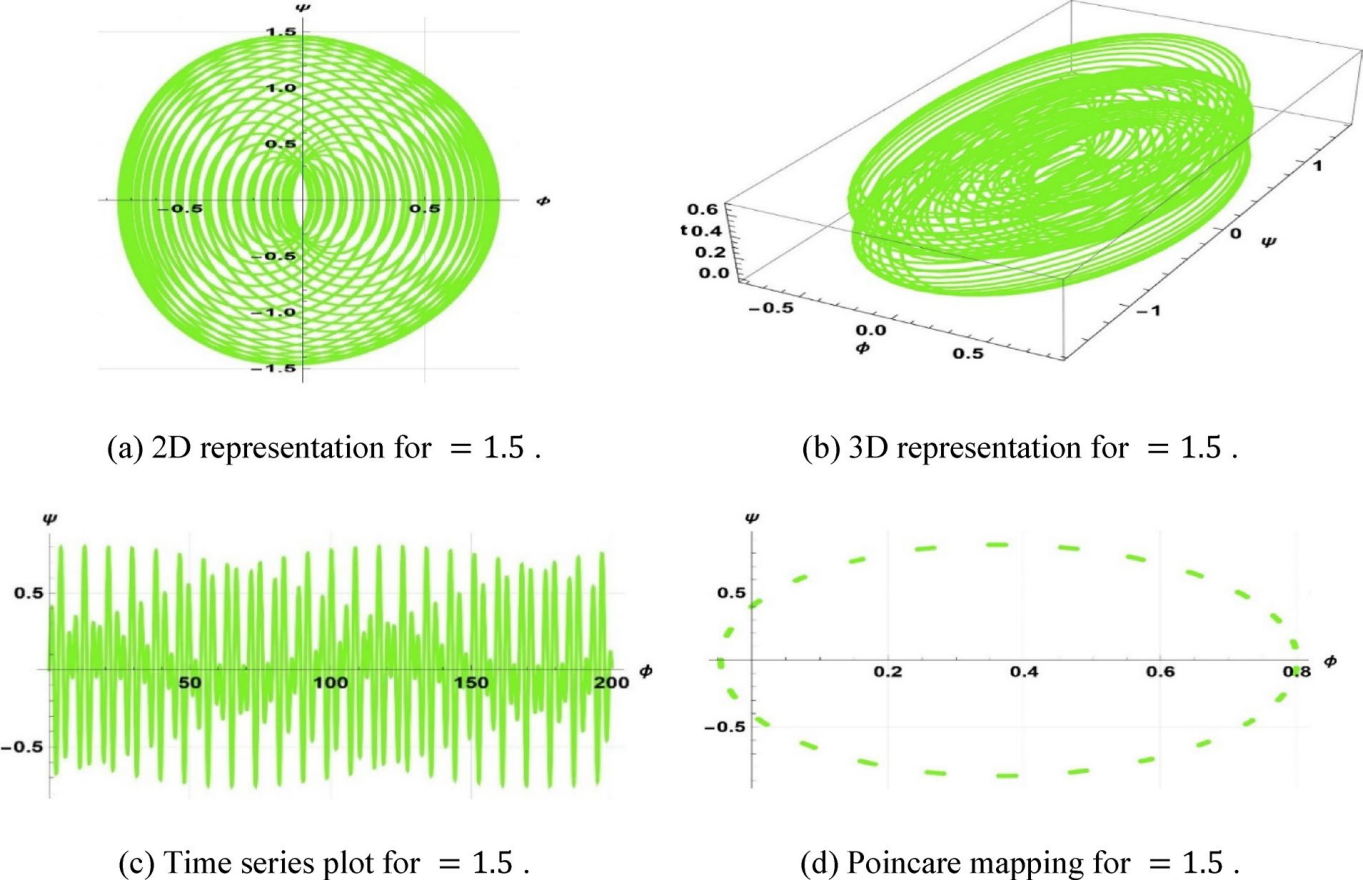
Illustration of Chaotic behaviors of system (8.1).

The system Eq. (8.1) shows as the effect of the perturbation term $\gamma cos(\eta t)$. In this system, $\gamma$ is the amplitude of external forcing, and the symbol $\eta$ indicates the frequency of terminal pull of the periodic perturbation. We will now examine a system as represented by Eq. (8.1), which illustrates a frequency-strength level multiplied by the specified levels of frequency and strength. Assuming that $k=5, \omega =2, a=2, b=1, c=3$, $\gamma =0.5, \& \eta =2.5$, the periodic patterns represented by Eq. (8.1) are depicted in Fig. 11. In the case of $k=3, \omega =2, a=3, b=2, c=5$, $\gamma =0.5, \& \eta =3.5$, the quasi-periodic character of Eq. (8.1) is illustrated in Fig. 12. In case $k=1, \omega =2, a=1, b=1, c=3$, ($\gamma =1, \& \eta =1.5$, Fig. 13 displays the chaotic behaviors of Eq. (8.1).

## Sensitivity analysis

Sensitivity analysis identifies a technique used to determine the effect of the uncertainties in the parameters of the input on the results of a mathematical model^57,60,60,71^. It contributes to determining what impacts productivity by models and how the variability of input is transmitted. This kind of analysis can help in establishing the strengths, stability, and quality of computational forecasts, thus making the decisions of complex systems with ease. The article discusses initial condition sensitivity to perturbed shape, where the sensitivity in mode periodicity does not vary with the amount of the perturbations, but high sensitivity is observed with low perturbation on the initial state of the perturbed system. The importance of the level of disturbance within the stability and dynamical reactivity of systems is noted by the study.

**Fig. 14:**
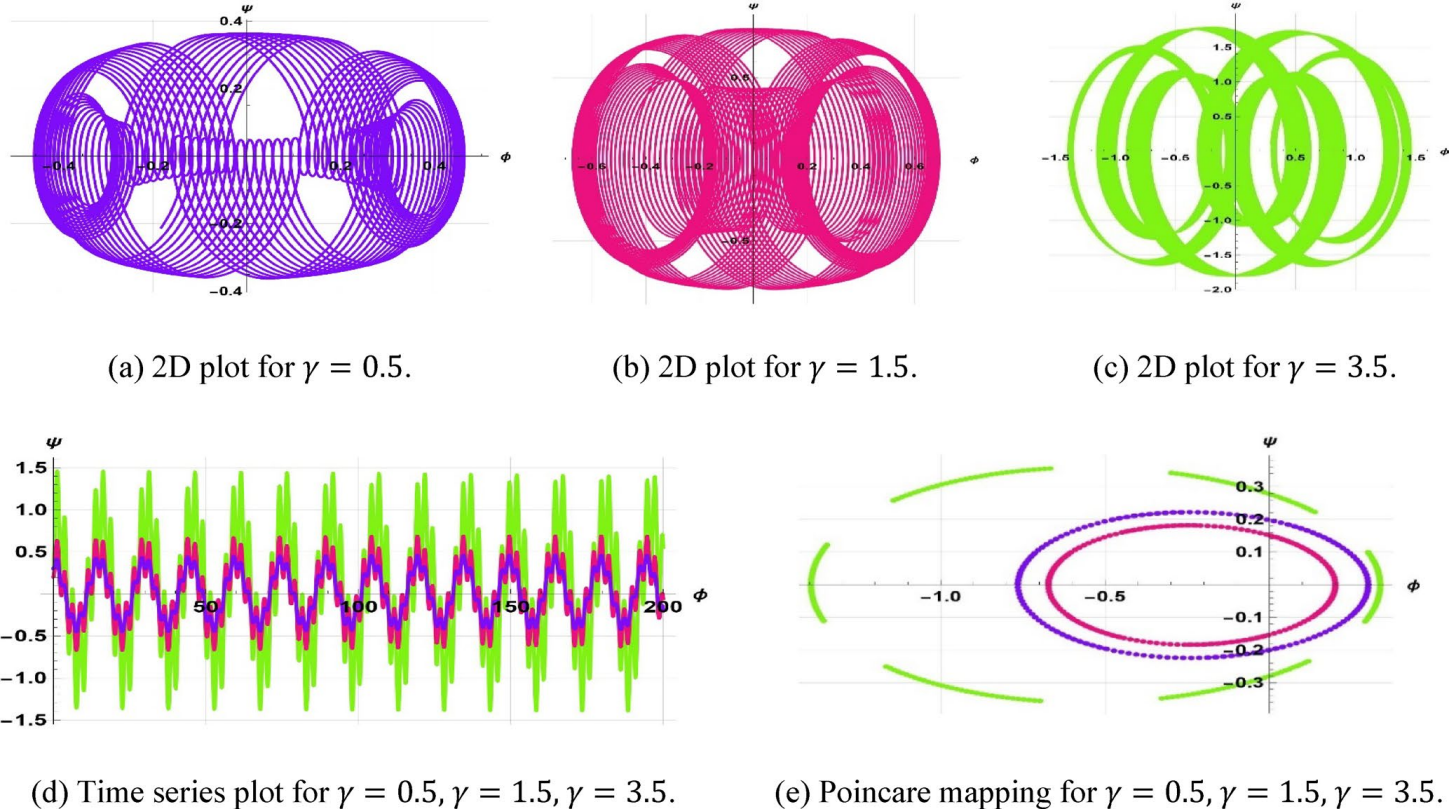
Visualization of sensitivity of (8.1) for $\gamma =0.5, \gamma =1.5, \gamma =3.5$.

The starting setup $\left(\mathrm{0.3,0}\right), \left(\mathrm{0.2,0}\right),\& (\mathrm{0.3,0.1})$, with parameters $k=5, \omega =2, a=2, b=1, c=3,$ and values $\gamma =0.5, \gamma =1.5, \gamma =3.5,$ and $\eta =2.5$, is illustrated in Fig. 14.

**Fig. 15:**
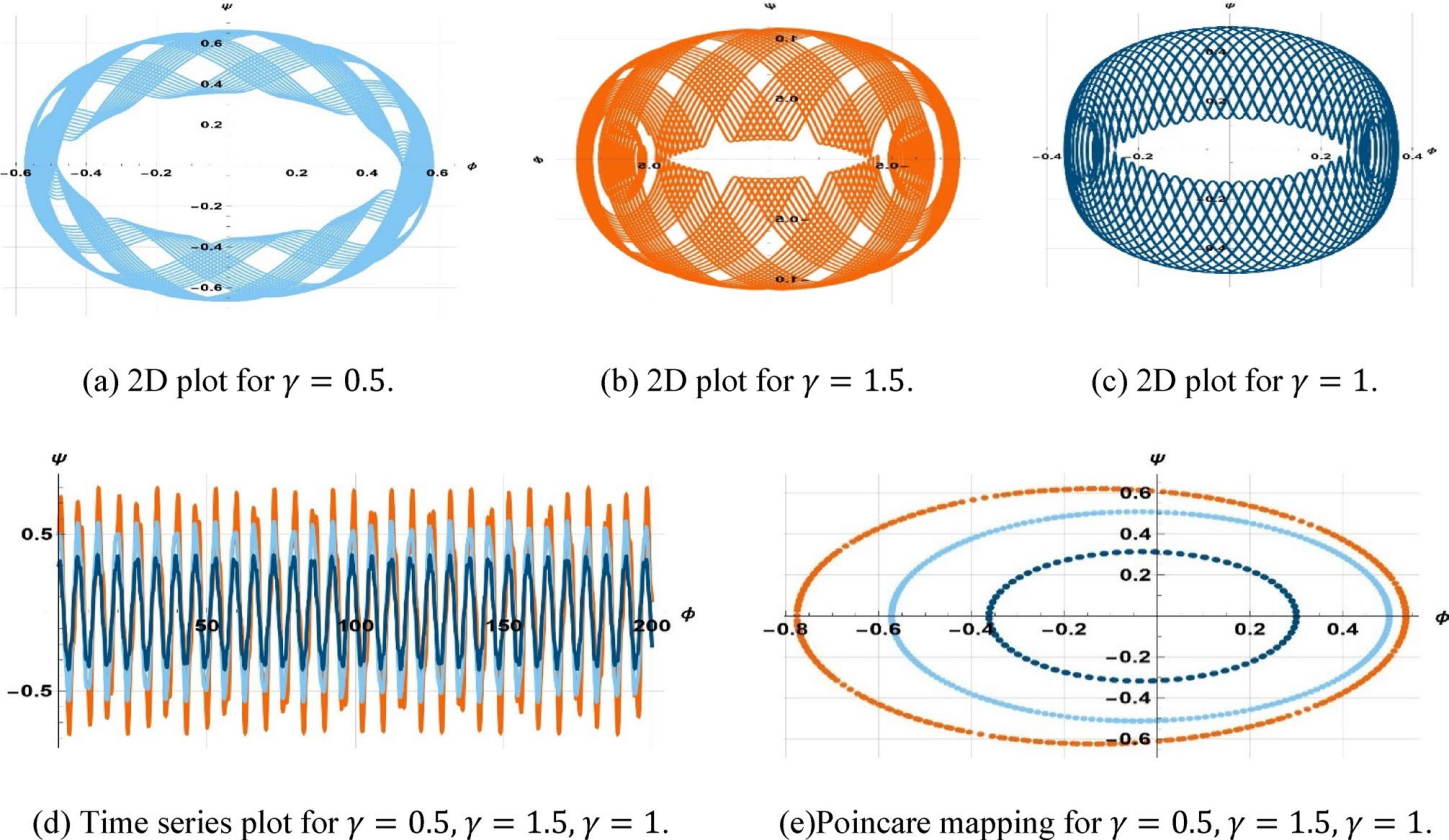
Visualization of sensitivity of (8.1) for $\gamma =0.5, \gamma =1.5, \gamma =1$.

The starting point $\left(\mathrm{0.5,0}\right), \left(\mathrm{0.5,0.2}\right),\& (\mathrm{0.3,0})$ and parameter values $k=3, \omega =2, a=3, b=2, c=5$, along with $\gamma =0.5, \gamma =1.5, \gamma =1$, and $\eta =3.5$, are illustrated in Fig. 15.

**Fig. 16:**
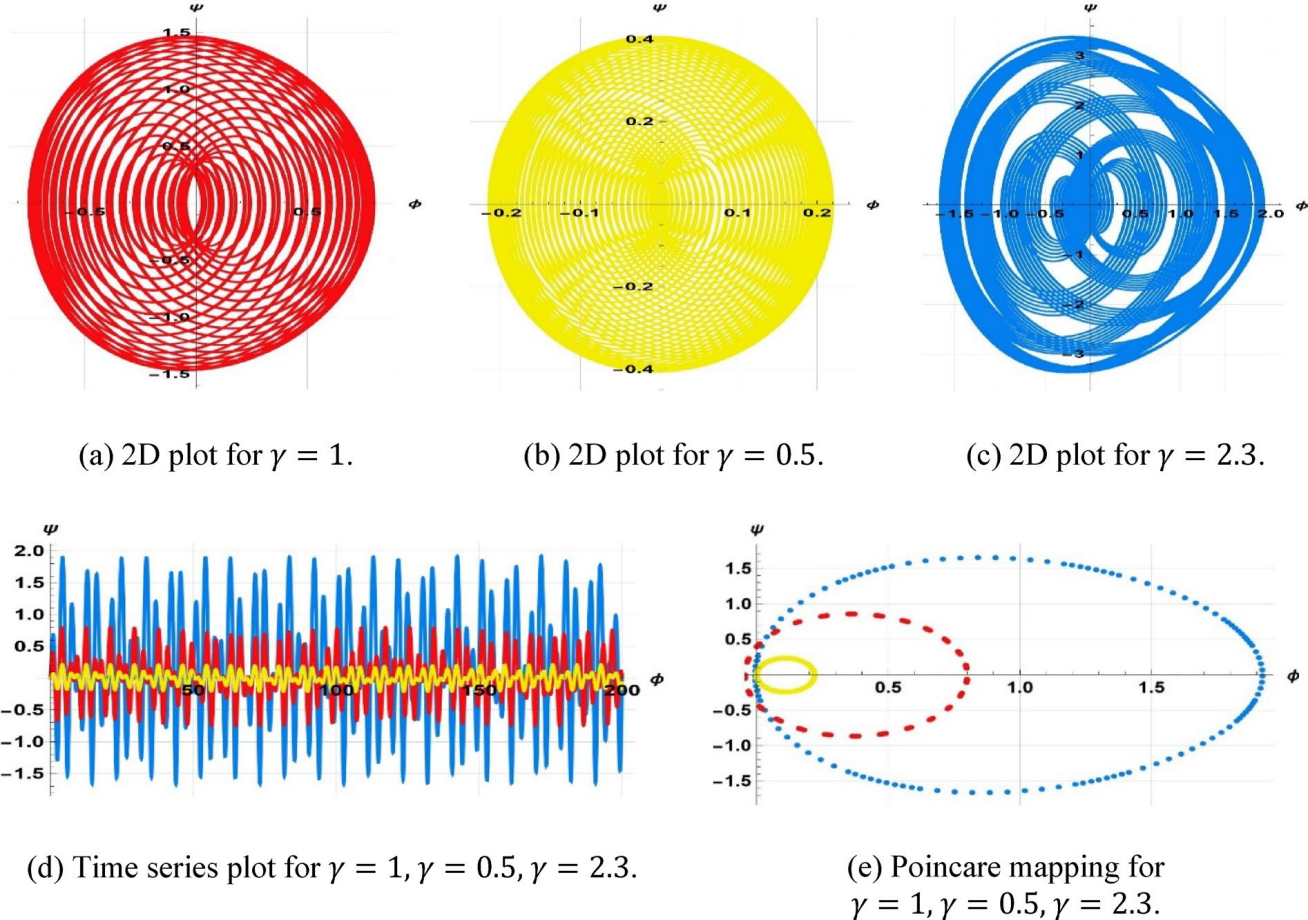
Visualization of sensitivity of (8.1) for $\gamma =1, \gamma =0.3, \gamma =2.3$.

Additionally, the starting configuration $\left(\mathrm{0,0.4}\right), \left(\mathrm{0,0}\right),\& (\mathrm{0,0.2})$, with parameter values $k=1, \omega =2, a=1, b=1, c=3$, ($\gamma =1, \gamma =0.3, \& \gamma =2.3$,), and $\eta =1.5$, is illustrated in Fig. 16.

## Lyapunov stability

**Fig. 17:**
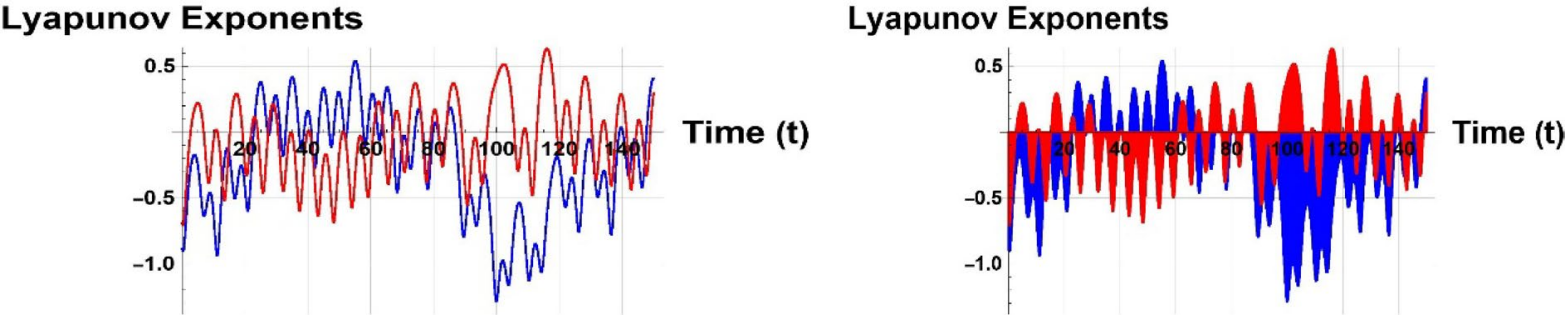
Representation of negative lyapunov stability in 2D patterns of (8.1).

Figure 17 displays chaotic systems with negative Lyapunov exponents, indicating their sensitivity to initial conditions. A negative Lyapunov exponent indicates dissipative dynamics or asymptotic stability in a dynamical system, implying infinitesimally close trajectories converge exponentially in time. This is applicable in non-chaotic regimes with stable fixed points, periodic attractors, or limit cycles.

## Novelty

The space–time fractional classical Boussinesq equation describes the nonlinear wave propagation in the thin water, plasmas, and lattices. This paper presents certain soliton solutions obtained using the modified extended tanh function technique, including bright and dark solitons, breather type, periodic waves, and $\mu -$ type soliton. Investigations on bifurcation, chaotic, sensitivity, and Lyapunov stability demonstrate the robustness and complex behavior of the system. The results highlight the uniqueness of the approach and suggest its potential application in various nonlinear waves. Comparing this method with other literature highlights its value in these fields, as shown in Table 1.

**Table 1:** Comparative analysis.

Study	Framework	Applied strategies	Acquired outcomes
Anwar Jaafar^15^	Cubic modified Boussinesq equation	The sine–cosine method	Dark solitons
Zhao et al.^42^	Boussinesq equation	Darboux and Backlund transformations	Exact solutions and continuum limit
Orkun et al.^35^	Two dimensional Boussinesq equation	Jacobi elliptic functions	New exact solutions
Current investigations	Space–time fractional classical Boussinesq equation	The modified extended tanh function method	Bright, dark, breather, periodic, & μ-type, soliton solutions; stability; Lyapunov stability; bifurcation and chaos graphical representation; sensitivity analysis

## Conclusion

This paper is a systematic analytical and dynamical analysis of the space–time fractional classical Boussinesq equation. With a better expansion scheme, we were able to achieve different soliton structures, such as bright, dark, breather-type, and periodic as well as μ-type waves and demonstrate how the evolution of these waves depends on the fractional order and system parameters. The bifurcation, phase-plane, and chaotic sensitivity analyses demonstrated how the system can exist in both stable and unstable states, as well as the high sensitivity of the initial conditions. The stability analysis in the form of perturbation validated the physical topicality of the solitons obtained. On the whole, the research provides a coherent conceptual framework for fractional nonlinear wave propagation and offers guidelines for predicting and controlling the waves. The directions taken in the future would be to include stochastic effects, study soliton interactions in various fractional orders, and prove the analytical results with numerical and experimental studies.

## Data Availability

The data used to support the findings of this study are included within the article.
